# Aberrant baseline brain activity in psychogenic erectile dysfunction patients: a resting state fMRI study

**DOI:** 10.1007/s11682-017-9805-9

**Published:** 2017-12-14

**Authors:** Chenwang Jin, Min Guan, Minghao Dong, Jia Wu, Zhen He, Xin Chen, Dapeng Shi, Junchan Ren, Guangming Shi, Xiangsheng Zhang

**Affiliations:** 1 Department of Medical Imaging, First Affiliated Hospital of Medical College, Xi’an Jiaotong University, Xi’an, China; 2 Department of Interventional Radiology, Henan Provincial People’s Hospital, Zhengzhou, 450003 China; 3 Engineering Research Center of Molecular and Neuro Imaging of Ministry of Education, School of Life Science and Technology, Xidian University, Xi’an, Shaanxi 710071 China; 4 Henan Andrological Academician Workstation of Basic and Clinical Research, Henan Provincial People’s Hospital, Zhengzhou, 450003 China; 5 School of Foreign Languages, Northwestern Polytechnical University, Xi’an, Shaanxi China; 6 Department of Urology, Henan Provincial People’s Hospital, Zhengzhou, 450003 China; 7 Department of Radiology, Henan Provincial People’s Hospital, Zhengzhou, 450003 China; 8 School of Electronic Engineering, Xidian University, Xi’an, Shaanxi, 710071 China

**Keywords:** Psychogenic erectile dysfunction, Resting state fMRI, Baseline Brain activity, ALFF, Experience

## Abstract

Recent neuroimaging studies have elucidated many interesting and promising findings on sexuality regarding the neural underpinnings of both normal and abnormal sexual processes. Psychogenic erectile dysfunction (pED) consists of a major part of male sexual dysfunction in China, but the understanding of the central mechanism of pED is still in its infancy. It is commonly appreciated that pED is a functional disorder, which can be attributed predominantly or exclusively to psychological factors, such as anxiety, depression, loss of self-esteem, and psychosocial stresses. Most previous studies probed the central response in the brain of pED patients using sexual-related stimuli. However, little concern has been given to a more fundamental issue whether the baseline brain activity is altered in pED or not. With rs-fMRI data, the current study aimed to explain the central mechanism behind pED by investigating the alterations in baseline brain activity in patients with pED, as indexed by the amplitude of low-frequency (0.01–0.08 Hz) fluctuation (ALFF). After the psychological screening and urological examination procedure, 26 pED patients and 26 healthy matched controls were enrolled. Our results explicated significantly lower baseline brain activity in the right anterior insula and right orbitofrontal cortex for pED patients (multiple comparison corrected). Additionally, the voxel-wise correlation analysis showed that ALFF of the right anterior insula was correlated with the outcomes of erectile function (multiple comparison corrected). Our results implied there was impaired cognitive and motivational processing of sexual stimuli in pED patients. Our current findings may shed light on the neural pathology underlying pED. We hope that our study has provided a new angle looking into pED research by investigating resting state brain activity. Furthermore, we suggest that the current study may put forward a more subtle conception of insular influence on pED, which may help foster new specific, mechanistic insights.

## Introduction

Psychogenic erectile dysfunction (pED) is defined as the persistent inability to achieve or maintain an erection adequate for copulatory behavior owing predominantly or exclusively to psychological or interpersonal factors, such as anxiety, depression, loss of self-esteem, and other psychosocial stresses (Wylie and MacInnes 2005). In China, epidemiology studies have shown that pED suffers consist of up to 90% of patients suffering from erectile dysfunction under their 40 s (Tan et al. 2007). Despite its high prevalence, the understanding of the central mechanism of pED is still in its infancy. Moreover, there is no effective treatment to pED yet due to the ambiguity in its underlying neural mechanism.

pED is independent of other vasculogenic or organic, neurologic, and endocrinologic mechanisms (Janssen 2007) and therefore is suggested to be a functional disorder, which can be attributed to abnormal central factors (Wylie and MacInnes 2005; Hatzimouratidis et al. 2010). Recently, neuroimaging studies have endeavored to disclose the neural mechanism underlying pED (Hagemann et al. 2003; Montorsi et al. 2003; Cera et al. 2012 and, 2014) and explicated spread brain areas, indicating the plausible role of high order factors in pED. Findings from our group (Zhang et al. 2014; Zhao et al. 2015a, b) and other groups (Cera et al. 2012) disclosed structural brain alterations in pED patients, such as the insula cortex, the orbital frontal cortex, the hypothalamus, the medial prefrontal, the cingulate cortex, and the inferotemporal cortex, which are also engaged in emotional and cognitive processing of sexual stimuli (Mouras, Stoléru et al. 2003; Redouté et al. 2005). In the absence of overt perceptual input and behavioral output, a restful brain consists of spontaneous fluctuations in neuronal activity (Zhang and Raichle 2010), as reflected in spontaneous activity of the blood oxygen level-dependent (BOLD) functional magnetic resonance imaging (fMRI). The resting state brain activity plays a pivotal role in maintaining the ongoing, internal representations (Lewis et al. 2009), which may be involved in the coding of expected sensory stimuli and prior experience (Miall and Robertson 2006). In other words, the resting brain is the collection of previous experience (Dong et al. 2014 and, 2015). Both typical and atypical sexual experiences modify human brain function, which in return influences subsequent sexual behavior (Georgiadis et al. 2012). Specifically, a previous conclusion demonstrated that pED is likely to derive from unsuccessful sexual experience caused by religious beliefs, interpersonal relationships, psychological well-being, and other factors (Wylie and MacInnes 2005). Also, rs-fMRI is used in previous studies in probing the central mechanism of a wide range of disorders (Barkhof et al. 2014; Satterthwaite and Baker 2015). Therefore, we suggest that resting state fMRI (rs-fMRI) may be capable of elucidating the underlying neural mechanism of pED (Hamaide et al. 2016).

The structural and task-based functional alterations in the brain of pED patients leads to an important question whether or not the baseline brain activity is altered in pED patients. We suggest that it be fundamental in that the changes in baseline brain activity may smear the spatial activation under task (Di et al. 2013), which may bring bias into previous conclusions. The amplitude of low frequency fluctuation (ALFF) is a reliable and reproducible method to measure baseline brain activity in both healthy participants (Yang et al. 2007; Zuo et al. 2010) and patients (Qi et al. 2012). Several studies employed it to measure the alterations in baseline brain activity for both healthy subjects (Zuo et al. 2010; Dong et al. 2015) and subjects with pathological conditions (Liu et al. 2012). Therefore, in the current study, we used ALFF as the metrics of baseline brain activity to determine whether or not the baseline brain activity was altered in pED patients. Given that empirical data and previous studies on pED reported no impairment in low-order processing (Cera et al. 2012, 2014; Zhao et al. 2015a, b), i.e. sensory and autonomic processing of the sexual stimulus, we expected to see baseline brain activity alterations in regions contributing to high-order mediation of erection, such as emotional, cognitive or motivational aspects (Stoléru et al. 2012). Furthermore, we conducted a voxel-wise correlation analysis to investigate whether or not such alterations were related to their behavioral measurements. We suggest that our current findings may shed light on the neural pathology underlying pED and would help foster new specific, mechanistic insights.

## Materials and methods

This study was carried out in accordance with the recommendations of the ethics committee of Henan Provincial People’s Hospital. The protocol was approved by the ethics committee of Henan Provincial People’s Hospital. All subjects gave written informed consent in accordance with the Declaration of Helsinki. The detailed study design was explained to all subjects and written informed consent was obtained from all subjects.

### Subjects

The subjects of the ED group were in-patients from the Department of Urology, Henan Provincial People’s Hospital. The subjects of the normal control group (NC) were recruited by advertisement. Sixty-four right-handed male heterosexual pED patients and fourty-one right-handed male heterosexual normal controls (NC) were recruited. After rigorous subject inclusion procedures (interview, physical examination and questionnaire evaluation), the remaining 52 subjects (26 pED subjects, 26.8 ± 5.0 years mean ± SD v.s. 26 NC subjects 28.5 ± 3.2years, mean ± SD) were recruited for the MRI scanning. All subjects were sexually experienced (Forbes et al. 2014).

In the initial interview session, the basic information (history of sexual relations, history of ED, demographic information, sexual experience, medical and medication history, surgical disorders, etc.) was collected by a doctor from the Department of Urology and confirmed by another doctor from the Department of Radiology respectively. Specifically, the medication history of subjects was carefully reviewed and recorded. Only subjects with a clear medication history remained in the subsequent procedure. Given that pED patients were in-patients with a history of ED for more than 6 months, it was especially difficult to recruit drug-naïve subjects. Therefore, we focused on patients whose medication history contained drugs with defined pharmacokinetic function, so that the MRI scanning could be conducted outside the washout period. According to records, patients included in this study took tadalafil, sildenafil, vardenafil and apomorphine. These drugs were easy to metabolize and caused no permanent effects on the subjects’ central nervous system (Altwein and Keuler 2001; Porst et al. 2001; Gaines 2004; Forgue et al. 2006). Subjects were asked to avoid taking these medicines one week ahead of MRI data acquisition to ensure they were free of the drug’s impact.

The exclusion criteria for both groups were: (1) use of any psychoactive medication and other medications that might affect sexual function; (2) history of using recreational drugs; (3) use of medication designed to enhance sexual performance; (4) history of committing any sexual offence; and (5) lacking experience of sexual intercourse. For the pED group, additional exclusion criteria were as follows: 1) history of ED less than six months. For the NC group, additional exclusion criteria were as follows: 1) history of ED.

The diagnosis of pED (generalized type) was performed according to current guidelines (Hatzimouratidis et al. 2010): absence of vasculogenic, neurogenic, hormonal, anatomical, and drug-induced ED. After an in-depth face-to-face interview, behavioral/physical assessments were carried out to exclude pED patients with vasculogenic or organic, neurologic, and endocrinologic factors. Normal morning erections, normal penile hemodynamics according to color Doppler sonography, and normal nocturnal erections were evaluated by the RigiScan device during three consecutive nights. These assessments were also conducted in the NC group, revealing no differences for both groups. Also, participants did not show any hormonal and metabolic disease.

### Behavioral/psychological measurement

Participants in this study also completed several questionnaires. The International Index of Erectile Function (IIEF) (Rosen et al. 1997) was used to assess erectile functioning for all participants. It is a 19-item questionnaire that requires men to provide Likert-scaled ratings of their experience with erectile function, intercourse satisfaction, orgasmic function, overall satisfaction, and sexual desire. The scales converge with clinical interviews, have high test–retest reliability, and do not co-vary with measures of social desirability (Rosen et al. 1997). It is a clinical and diagnostic evaluation of ED severity. IIEF is not a useful tool for differentiating vascular causes of erectile problems (Kassouf and Carrier 2003), but it has been used in thousands of studies to quantify clinical improvement (Martin-Morales et al. 2014) and general erectile functioning (Janssen et al. 2014). Furthermore, Self-Rating Anxiety Scale (SAS) (Jegede 1977) and Self-Rating Depression Scale (SDS) (Zung 1965) were used to evaluate the anxiety and depression levels for all participants involved.

### MRI data acquisition

Magnetic resonance imaging (MRI) data in this study were obtained on a 3T GE 750 Discovery MRI scanner (General Electric, Milwaukee, WI, USA), with a dedicated 8-channel head coil. Resting-state functional images were collected using an echo-planar imaging sequence with the following parameters: scan duration = 8 min, repetition time = 2000 ms, echo time = 30 ms, field of view = 24, flip angle = 90°, number of slices = 240, slice thickness = 4 mm, gap = 0 mm, and matrix = 64 × 64. During the whole scanning process, the subjects were asked to keep their eyes closed, their head still and stay awake. After the scanning session, the subjects were asked whether they had fallen asleep during the scanning.

### Functional data preprocessing

Data preprocessing procedures were carried out using the DPABI toolbox v2.0 (http://rfmri.org/dpabi) and SPM 8 (http://www.fil.ion.ucl.ac.uk/spm) under Matlab2009a.

#### Resting data processing

For each subject, the first 10 volumes were discarded to eliminate non-equilibrium effects of magnetization and allow the participants to adapt to the EPI scanning environment. The images were corrected for the acquisition delay between slices, motion corrected by regressing out the six rigid body parameters, and co-registered to the subject’s anatomical images in native space. No subjects had head motions exceeding 1.5 mm of movement or 1.5°of rotation in any direction. The nuisances (head motion, white matter and cerebro-spinal fluid signals) were regressed out. Next, all functional images were normalized to MRI space using the deformation field maps obtained from structural image segmentation following the segmentation routine in SPM8. The normalized images were resampled to 3 mm isotropic voxels, which were then spatially smoothed with a 6 mm full width at half maximum Gaussian kernel. Finally, linear trend was removed and temporal filtering (0.01–0.08 Hz (Biswal et al. 1995)) was performed on the time series of each voxel to reduce the effect of low-frequency drifts and high-frequency noise.

#### ALFF map

The ALFF analysis was carried out using the REST package (http://resting-fmri.sourceforge.net), which has been described in previous studies (Yang et al. 2007). Briefly, the filtered time series was transformed to the frequency domain using the fast Fourier transform (FFT). Since the power of a given frequency was proportional to the square of the amplitude of this frequency component, the square root was calculated at each frequency of the power spectrum and the averaged square root was obtained across 0.01–0.08 Hz at each voxel. This averaged square root was taken as the ALFF. For standardization, the ALFF of each voxel was divided by the global mean ALFF value for each subject, resulting in a relative ALFF, the standardized ALFF of each voxel then had a value of about 1, as done in PET studies (Raichle, MacLeod et al. 2001).

### Statistical analysis

#### Between-group ALFF analysis

For the between group analysis, a two-sample *t*-test was performed to detect the ALFF difference between the two groups. The results were considered significant above a threshold of *p* < *0.05 (family-wise error, FWE corrected)*.

#### Correlation analysis

To examine the relationship between the behavioral/psychological measurement and the brain measurement, voxel-wise correlation analysis was performed between the duration of pED, scores of IIEF, SAS, SDS and ALFF outcomes. The results were considered significant above a threshold of *p* < *0.05 (family-wise error, FWE corrected)*.

#### Post hoc analysis

In the present samples, SAS and SDS scores showed significant differences between the two groups. Therefore, we repeated the between-group comparison in the FC analysis after controlling SAS and SDS scores to ensure that these variables were not driving our results.

## Results

### Results of demographic and psychological/behavioral data analysis

As shown in Table 1, pED and NC groups were not significantly different from the NC group in terms of age and level of education (*p* > *0.05, two-sample t-test, two-tailed*). pED patients had lower IIEF scores (*p* < *0.05, two-sample t-test, two-tailed*), indicating impaired erectile function. As expected, the pED group also had higher SAS (*p* < *0.05, two-sample t-test, two-tailed*) and SDS scores (*p* < *0.05, two-sample t-test, two-tailed*) than NC. These findings are not surprising in that established clinical evidence reported high prevalence of negative psychological effects, such as anxiety, depression and embarrassment, in men with impaired sexual function (Janssen 2007; Hatzimouratidis et al. 2010).

**Table 1:** Demographics and behavioral measurements in the pED group and the healthy control group

	pED (n = 26)		NC (n = 26)		Two sample T Test (two tailed)
	Mean	SD	Mean	SD	*p* value
Demographic Data					
Age (years)	26.8	5.0	28.5	3.2	0.19
Education (years)	13.6	1.8	14.4	2.1	0.17
Behaviral data					
IIEF-total*	30.9	8.7	64.1	5.1	< 0.001
SAS*	45.3	7.0	39	7.9	0.025
SDS*	49.3	8.3	39	8.1	< 0.001

pED: Psychogenic erectile dysfunction; NC: Normal controls; SD: Standard deviation; IIEF: International Index of Erectile Function; SAS: Self-rating anxiety scale; SDS: Self-rating depression scale

^*^Denotes significant difference between groups (*p* < *0.05*)

### Results of between-group ALFF results

Compared with NC, the results of the two-sample *t*-test demonstrated a significantly lower ALFF value (*p* < *0.05, voxel level, FWE corrected, pED v.s. NC*) in the right anterior insular cortex (AI) and the right orbitofrontal cortex (OFC) in pED patients, indicating a significantly lower baseline brain activity in the pED group. The localization of the anterior insular cortex was based on the method and templates proposed in a previous publication (Kelly et al. 2012). No brain regions with a significant ALFF increment were found in the pED group.

### Results of the correlation analysis

No significant correlations or negative correlations were identified at the threshold of *p* < *0.05 (FWE corrected)*. Therefore, a secondary but rigorous threshold of *p* < *0.01 (AlphaSim corrected)* was adopted. Voxel wise correlation analysis demonstrated a significant correlation between baseline brain activity in the right AI, as indexed by ALFF, and erectile functioning, as indexed by IIEF scores (*Alphasim correction, p* < 0.01 at cluster level, the corresponding statistical level is set at |Z | >3.33 (*p* < 0.001 at voxel level, 5000 times of the Monte Carlo simulations using the AFNI AlphaSim program) and cluster size > 124 voxels (*search volume* = *70,831 voxels*)), which corresponds to a corrected *p* < *0.01* at cluster level (Fig. 2a). Its peak voxel (34, 14, 4; Z = 6.0) was located within the right AI. We displayed a scatter plot between IIEF ratings and the peak ALFF voxel from the correlation map (*r* = 0.72, *p* = 1.76 × 10^− 9^) for the purpose of illustration, otherwise it would be a circular analysis (Fig. 2b). No such correlations or negative correlations were found between outcomes of other behavioral tests (SAS, SDS) and ALFF, as well as between the duration of pED and ALFF for the pED group exceeding the same level of significance.

### Results of post hoc analysis

The results were not changed after controlling SAS scores and SDS scores, indicating that the outcomes reported in the current study were not driven by SAS/SDS differences. Established clinical evidence reported high prevalence of negative psychological effects, such as anxiety, depression and embarrassment, in men with impaired sexual function (Janssen 2007 and; Hatzimouratidis et al. 2010), suggesting that these factors are inseparable from the pED study. Therefore, we proposed that slight anxiety/depression should be taken as the net effect of pED. Accordingly, please note that the major findings of the current study were outcomes without controlling SAS and SDS as covariates.

## Discussion

**Fig. 1:**
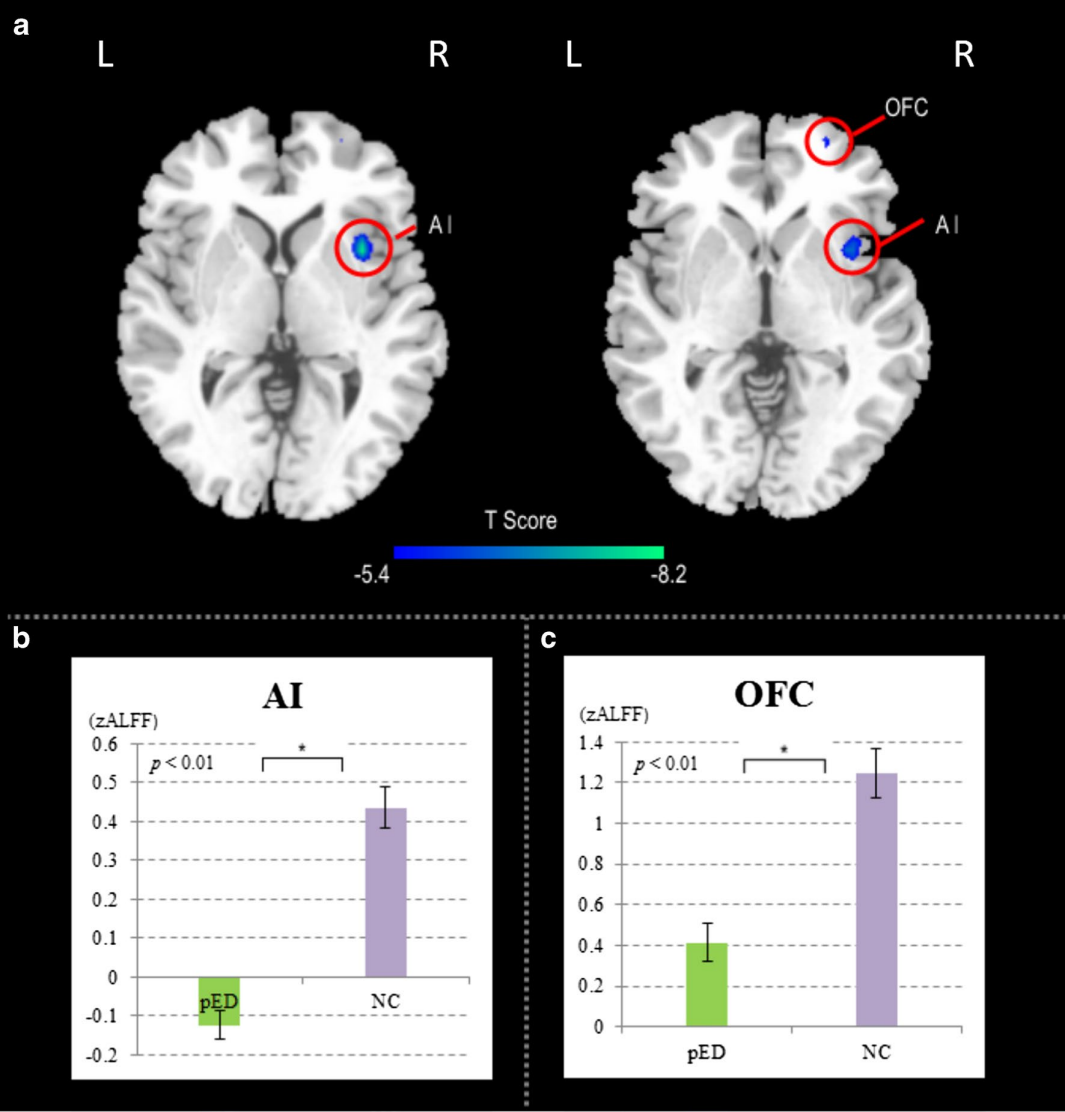
Results of ALFF differences between the pED group (*n* = 26) and healthy control group (*n* = 26) (*p* < 0.05, *FWE* corrected, voxel level, displayed in axial view, *pED v.s. NC*). Error bars: *SEM*. (**a**) A significantly lower baseline brain activity of the right AI and OFC in pED patients was observed as compared with normal controls. (**b**) baseline brain activity averaged within regions which showed significant ALFF differences between groups, i.e. right AI (**b**) and OFC (**c**). *Indicates significant group differences (*p* < 0.01)

**Fig. 2:**
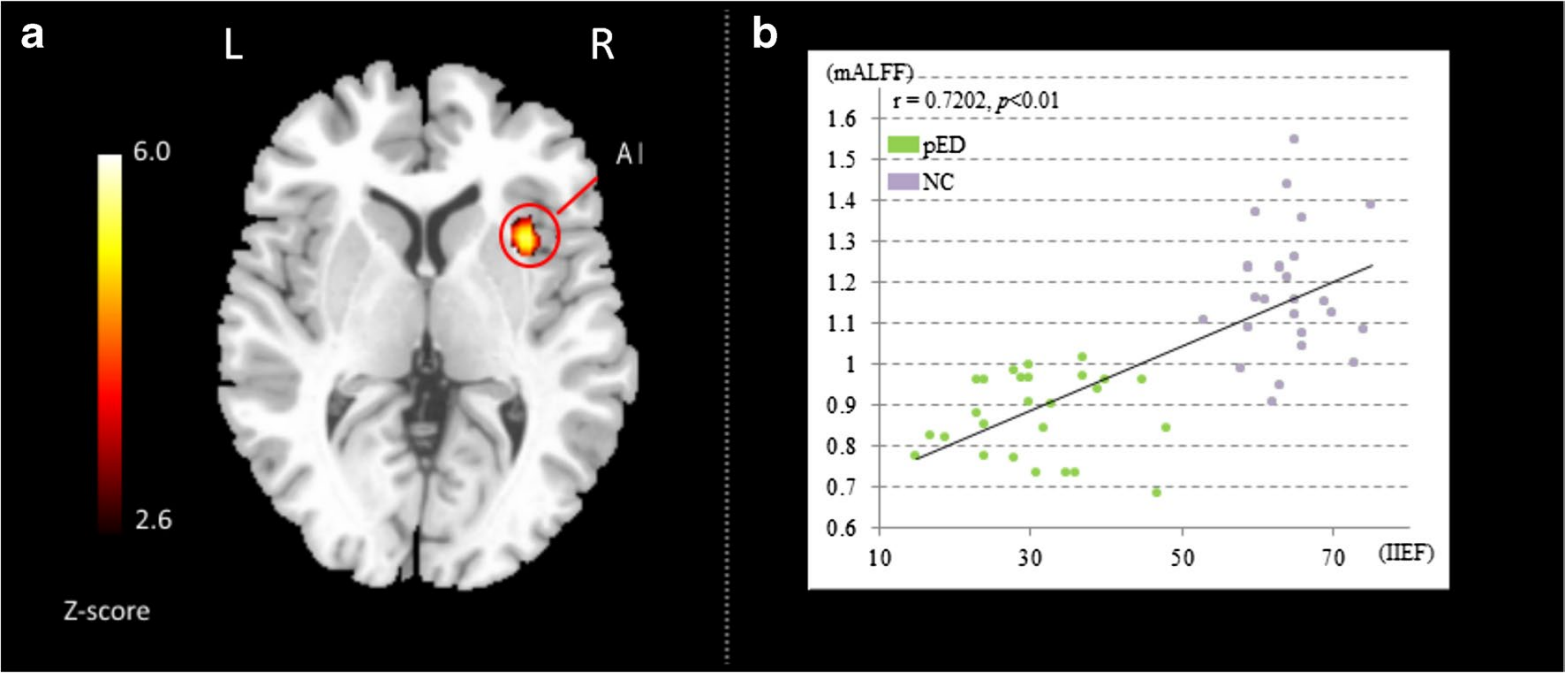
Voxel-wise correlation map between baseline brain activity as indexed by ALFF and erectile functioning as indexed by IIEF scores. Error bars: *SEM*. (**a**) Significant correlation was identified in the right anterior insula shown between ALFF and IIEF in all subjects (n = 52, *p* < 0.01, Alphasim corrected). The statistical threshold was set at |Z| > 3.33 (*p* < 0.001 at the voxel level) and cluster size > 124 voxels, which correspond to a corrected *p* < 0.01 at the cluster level. (**b**) The scatter plot map computed as ALFF of the peak voxel (34, 14, 4) in the correlation analysis and IIEF scores. Please note that this map is only for illustration purposes, otherwise there would be the risk of a circular analysis

Psychogenic erectile dysfunction (pED) is a functional disorder (Wylie and MacInnes 2005) and previous fMRI studies investigating the central mechanism of pED used visual sexual stimuli (Cera et al. 2012 and, 2014). The current study focused on resting state fMRI (rs-fMRI) and investigated a fundamental issue in pED studies whether or not the baseline level brain activity, as indexed by ALFF, was altered in pED. Specifically, for the behavioral data, pED showed impaired erectile function compared with NA, as depicted by lower IIEF scores (Fig. 1; Table 2). For the MRI data, significantly lower ALFF was identified in the right anterior insula (AI) and the right orbitofrontal cortex (OFC) in the pED group. The level of erectile function was correlated with baseline brain activity in the right AI using a voxel-wise correlation analysis (Fig. 2; Table 3). We suggest that our current study may shed new light on the neural mechanism of pED.

**Table 2:** Significant group ALFF differences (*p*_FWE_<0.05) between the pED group (n = 26) and the healthy control group (n = 26)

	**Hemisphere**	BA	MNI Coordinates (cluster maxima)			Voxels	t (cluster maxima)
			**x**	**y**	**z**		
AI	R	-	36	9	0	34	-8.2
OFC	R	11	25	57	-3	10	-5.8

AI: anterior insula; OFC: orbitofrontal cortex; R : right ; BA : brodmann area;

**Table 3:** Significant voxel-wise correlation between ALFF and all the subjects (n = 52) (*p* < 0.01, Alphasim corrected)

	Hemisphere	BA	MNI Coordinates (cluster maxima)			Voxels	t (cluster maxima)
			**x**	**y**	**z**		
AI	R	-	34	14	4	124	6.0

AI: anterior insula; R : right ; BA : brodmann area;

### Resting state fMRI and pED

The current study uniquely focused on the resting state brain activity rather than brain responses under stimulation of sexual stimuli. We argue that rs-fMRI is of great importance to pED studies.

Firstly, we suggest that baseline issue is rudimentary in fMRI studies, particularly for studies using task paradigms, in that (1) Changes in baseline brain activity may smear the spatial activation under task (Di et al. 2013); (2) Baseline alterations may bring biased results into the fMRI study (Lee et al. 2013 and; Dong et al. 2015). This issue is more urgent given that a major part of existing MRI literature on pED central mechanism used tasks paradigms (Cera et al. 2012 and, 2014). Therefore, we started from this fundamental issue and investigated the baseline brain activity changes using rs-fMRI. Secondly, to a fair extent, sexuality is a learned process (Georgiadis et al. 2012). Even the quality of sexual activity (Pfaus et al. 2012) contributes to sexual learning (Georgiadis et al. 2012). And, the learning process shapes or modifies human brain function with respect to components of sexual activity, which in return influencing subsequent sexual behavior both typically and atypically(Georgiadis 2012). In other words, all mismatched failure experiences are associative with learning process and written into the brain, which may explain how sexual difficulties or even dysfunctions develop (Both et al. 2008; Hoffmann et al. 2012; Woodson 2002). Clinical observations suggested that the erectile dysfunction may develop from unsuccessful experience (Rosen 2001). Resting state actively processes prior experience (Miall and Robertson 2006), which may facilitated in the coding learned experience (Peigneux et al. 2006). Therefore, resting state data may conceive important information regarding the central mechanism of pED. Last but not least, sexual responses are induced by both external stimuli, encompassing all sensory modalities, such as visual input (Stoleru et al. 1999; Redouté et al. 2000; Bocher et al. 2001; Arnow et al. 2002; Karama et al. 2002; Hagemann et al. 2003), tactile input (Holstege et al. 2003; Georgiadis et al. 2007), auditory input (Georgiadis et al. 2012) and olfactory input (Huh et al. 2008; Pfaus et al. 2012), and even internal stimuli which is independent of any sexual bodily stimulation, i.e., sheer mental force (e.g. imagery) (Smith and Over 1991; Whipple et al. 1992; Beauregard et al. 2001). Empirical data and previous studies on pED report no impairment in the low order processing of sexual stimulus(Cera et al. 2012, 2014; Zhao et al. 2015a, b), i.e. sensory and autonomic information, thus suggesting that the high order processing of sexual stimulus, i.e. cognitive, emotional, anamnestic and motivational aspects, may be problematic. These processes are independent of sensory input modality, therefore, the rs-fMRI, which is absent of sensory stimulation, could enable us to focus on the core process of sexual stimuli processing.

### The baseline alteration in the right anterior insula (AI)

The current study reported baseline disruption in the right AI, whose level of baseline brain activity was also correlated with erectile dysfunction scores (Fig. 1a, b; Table 2). The insula is hard-wired in a sexual cycle, especially in the mediation of erection. Supportive evidence came from animal studies using rodents, given the homology between the human and rodent insula in terms of corresponding cytoarchitecture and connectivity (Namkung et al. 2017). Particularly, the insula is activated by copulation with a preferred scented partner (Pfaus and Heeb 1997) and mediation of erectile function in male rats (Georgiadis 2012; Georgiadis et al. 2012). In neuroimaging studies involving healthy males, the activation of the insula has been consistently reported (Stoleru et al. 1999; Stoleru 2014; Sescousse et al. 2013) especially its direct role in mediation of penile erection, such as perceptual processing of penile inputs (Moulier et al. 2006), onset of erection (Miyagawa et al. 2007), recognition of erection (Arnow et al. 2002) and sustained penile response to erotic stimuli (Ferretti et al. 2005). In contrast, the malfunction of the insula was reported in subjects with male sexual dysfunction (Stoléru et al. 2003; Gratzke et al. 2010), particularly in pED studies (Cera et al. 2012, 2014; Zhao et al. 2015a, b). Moreover, in the current study, the erection scores of subjects were correlated with baseline activity of the right AI, confirming its important role in the mediation of erection.

Indeed, erection is the anchor of male sexual activity. It is the production of highly dynamic interplay of central/supraspinal signaling and periphery information (Giuliano and Rampin 2000; Andersson 2011). The process engages complicated components, such as perceptual, cognitive, motivational, emotional, inhibitory and autonomic components, encompassing a network of brain areas (Georgiadis et al. 2012; Poeppl et al. 2014; Zhao et al. 2015a, b). Erection is invariably associated with physiological states, most importantly the change in genitals, which gives rise to strong interoceptive signals (Andersson 2011). Basically, interoceptive signals arrive in the posterior insula where low-level sensory features are processed (Namkung et al. 2017). Then, AI integrates the re-represented interoceptive signals passed on from the posterior insula with emotional, cognitive, and motivational signals collected from other cortical and subcortical regions (Gasquoine 2014; Droutman et al. 2015). Aberrant baseline brain activity in the right AI may indicate functional abnormality or deterioration in this area, although its exact biological basis is to be determined. Given that AI has a central role in supporting subjective feeling states, as well as regulating the introduction of insula-mediated feelings into cognitive and motivational processes in behaviorally relevant contexts (Gasquoine 2014), three main interpretations regarding insular involvement in pED can be proposed. Firstly, dysfunction of the AI is mirrored in abnormal subjective feelings (Craig 2009) leading to biased encoding in sexual context. Rewarding sexual stimuli normally evoke feeling of pleasure, but, in pED, it is quite likely that sexual stimuli are represented as aversive feelings, eventually leading to failure of engagement in copulatory behaviors. Secondly, AI plays an important role in cognition (Namkung et al. 2017): sexual stimulation is highly salient and AI initiates cognitive processes for processing salient information by marking salient information, i.e. sexual stimuli. Therefore, dysfunction of AI may prioritize cognitive resources for other extractions other than sexual stimuli, eventually leading to inability of maintaining erection. Thirdly, AI plays an important role in motivation (Naqvi and Bechara 2009), i.e. subject desire to engage in behaviors. Converging evidence suggests that the insula encodes incentive values of stimuli by evaluating the stimuli-eliciting subjective feeling states (Balleine and Dickinson 2000; Naqvi and Bechara 2009). Dysfunction in the AI may disrupt the encoding of sexual stimuli. In other words, sexual stimuli, which are rewarding, evoke feelings of pleasure and in turn drive conscious desires for deciding to take particular actions, may be regarded as aversive stimuli in pED and thus lead to conscious aversions for avoiding engagement in sexual behaviors. Indeed, our interpretation of this finding can hardly be conclusive until our follow-up studies are better tailored to such specific interpretations delivering more satisfactory answers.

### The baseline alteration in the right OFC

The current study reported decreased baseline activity in the right OFC (Fig. 1a, c; Table 2). A number of lesion, electrophysiological, and neuroimaging studies indicated a general role for the OFC in encoding the value assigned to different goods, including sexual rewards, pleasant touch, pleasant odors, or visual presentation of food in both human (Plassmann et al. 2007; Chib et al. 2009; FitzGerald et al. 2009; Sescousse et al. 2010) and nonhuman primates (Padoa-Schioppa and Assad 2008). In the case of sexual stimuli, the right OFC is identified as the neural correlate of the process of appraisal through which stimuli are categorized as sexual incentives and quantitatively evaluated as such (Redouté et al. 2005), and thus eventually assigning motivational significance to sensory inputs(Stoléru et al. 2012). We suggest that the observed lower baseline brain activity in the pED group may indicate impaired processing of cognitive/motivation processes of sexual incentives(Namkung et al. 2017), which would jeopardize male sexual arousal. Given the reciprocal connections between the OFC and AI (Droutman et al. 2015), the dysfunction in the right AI and OFC may act upon each other, giving rise to incorrect interpretation of sexual incentive. This may cause a long-term deficit in sexual reward, which is a major factor for sexual learning. Eventually, atypical sexual learning may be closely related to pED.

## Limitations

We suggest that several limitations should be taken into consideration when interpreting the current findings. Firstly, the sample sizes were relatively small after the rigorous subject exclusion process. Although the results survived a strict statistical threshold, we still suggest that the findings should bear replication in other studies using larger samples. Actually, one major aim of our currently undergoing study is to verify the reliability of our findings. Secondly, an individual’s sexual response is the net outcome of many factors. These include factors that have already been controlled in the current study and those beyond our research, such as genetic or innate (e.g., gender) factors, or factors related to transient changes of the internal state induced by stress, metabolic state or gonadal hormones (Georgiadis and Kringelbach 2012; Pfaus et al. 2012; Stoleru et al. 2012). However, this is a general issue encountered by any other cross-sectional experiment. We are now looking at the chance to conduct a longitudinal study to solve this issue, which is rather challenging. Thirdly, in studies using patients, drug-naïve subjects are most preferable. In the current study, we did not use subjects in this category, because they are so scarce, especially for patients suffering from pED for at least six months. Alternatively, we controlled the washout period of the drug and strictly made sure that the drugs exerted no influence on the central nervous system during MRI scanning. Moreover, we suggest that these patients should be of great value and interest for clinical neuroscience in that the patients with medication history, rather than drug-naïve state, are the real-life case scenario for most clinical studies.

## Conclusion

The current study stemmed from resting state fMRI and investigated a fundamental issue in pED neuroimaging studies. Our results suggested that the deficit in erectile functioning was mirrored in aberrant baseline brain activity in the right AI and the right OFC, which may imply there is an impaired cognitive and motivation component of sexual stimuli processing in pED patients. In particular, our results highlighted the crucial role of the right AI in the pathophysiology of pED; our follow-up study would investigate how the brain network which is anchored in this region is distinguished from that of healthy controls. Furthermore, our study may put forward a more subtle conception of insular influence on pED, which would help foster new specific, mechanistic insights. Last but not least, by introducing sexuality from a neuroplastic perspective, we suggest that our study has provided a new angle exploring the neural substrates of pED and may shed light on the neural pathology underlying pED.

## Abbreviations

rs-fMRI: Resting state functional magnetic resonance imaging
ED: Erectile dysfunction
pED: Psychogenic erectile dysfunction
NC: Normal control
AI: Anterior insular
OFC: Orbitofrontal cortex
IIEF: The International Index of Erectile Function
SAS: Self-Rating Anxiety Scale
SDS: Self-Rating Depression Scale
SD: Standard deviation
SEM: Standard Error of the Mean
